# Peripheral and Central Mechanisms of Persistent Orofacial Pain

**DOI:** 10.3389/fnins.2019.01227

**Published:** 2019-11-13

**Authors:** Masamichi Shinoda, Asako Kubo, Yoshinori Hayashi, Koichi Iwata

**Affiliations:** Department of Physiology, Nihon University School of Dentistry, Tokyo, Japan

**Keywords:** orofacial ectopic pain, trigeminal ganglion, trigeminal spinal subnucleus caudalis and upper cervical spinal cord, satellite cell, macrophage, microglia, astrocyte

## Abstract

Neuroplastic changes in the neuronal networks involving the trigeminal ganglion (TG), trigeminal spinal subnucleus caudalis (Vc), and upper cervical spinal cord (C1/C2) are considered the mechanisms underlying the ectopic orofacial hypersensitivity associated with trigeminal nerve injury or orofacial inflammation. It has been reported that peripheral nerve injury causes injury discharges in the TG neurons, and a barrage of action potentials is generated in TG neurons and conveyed to the Vc and C1/C2 after trigeminal nerve injury. Long after trigeminal nerve injury, various molecules are produced in the TG neurons, and these molecules are released from the soma of TG neurons and are transported to the central and peripheral terminals of TG neurons. These changes within the TG cause neuroplastic changes in TG neurons and they become sensitized. The neuronal activity of TG neurons is further accelerated, and Vc and C1/C2 neurons are also sensitized. In addition to this cascade, non-neuronal glial cells are also involved in the enhancement of the neuronal activity of TG, Vc, and C1/C2 neurons. Satellite glial cells and macrophages are activated in the TG after trigeminal nerve injury and orofacial inflammation. Microglial cells and astrocytes are also activated in the Vc and C1/C2 regions. It is considered that functional interaction between non-neuronal cells and neurons in the TG, Vc, and C1/C2 regions is a key mechanism involved in the enhancement of neuronal excitability after nerve injury or inflammation. In this article, the detailed mechanisms underlying ectopic orofacial hyperalgesia associated with trigeminal nerve injury and orofacial inflammation are addressed.

## Introduction

Trigeminal nerve injury and orofacial inflammation are known to frequently cause persistent pain that can spread to adjacent orofacial regions innervated by the uninjured trigeminal nerve branches. Peripheral and central mechanisms are considered to be involved in the persistent ectopic orofacial pain associated with trigeminal nerve injury or orofacial inflammation (Imbe et al., 2001). Orofacial ectopic pain is defined as pain spreading from injured branch regions to uninjured branch areas following trigeminal nerve injury or orofacial inflammation. Orofacial ectopic persistent pain is sometimes difficult to diagnose and treat. After tooth extraction or tooth-pulp inflammation, ectopic persistent pain occurs in the uninjured and non-inflamed areas of the orofacial regions (Chiang et al., 2011).

Following trigeminal nerve injury, primary afferent neurons are significantly activated, and a barrage of action potentials is generated in TG neurons and conveyed to the central nervous system. Background activity is also augmented in TG neurons, resulting in the increment of baseline activity in the primary afferent neurons. nNOS expression in the sensitized primary afferent neurons accelerates NO synthesis, and NO is released from the sensitized primary afferent neurons. Neuronal excitability in the uninjured trigeminal nerve branches is altered by NO signaling, the injured neurons and increased excitability of uninjured neurons together contribute to persistent pain (Sugiyama et al., 2013). The soma of a primary afferent neuron is known to be tightly surrounded by SGCs in the TG. Connexins are the primary components of gap junctions organized into two hemichannels called connexons and contribute to binding SGCs. Connexin 43 (Cx43), which is the primary gap junction protein, is known to modulate transportation of small molecules between SGCs. Following trigeminal nerve injury, SGC activation via gap junctions composed of Cx43 propagates throughout the TG, resulting in the sensitization of uninjured TG neurons responsible for ectopic orofacial pain (Kaji et al., 2016). The gap between the soma of primary afferent neurons and the SGCs is only 20 nm, and these cells communicate with each other by releasing chemical messengers. Various molecules are released from TG neurons and cause activation of SGCs, and activated SGCs also generate many molecules that are released from SGCs in the TG, leading to acceleration of TG neuronal activity (Chiang et al., 2011).

Non-neuronal cells such as macrophages are accumulated in the TG after trigeminal nerve injury and orofacial inflammation. Two types of macrophages, M1 and M2, are activated and infiltrate in the injured and inflamed sites and the TG. Macrophages release various cytokines and chemokines, and these molecules are involved in the modulation of TG neuronal activity. M1 and M2 are known to be differentially involved in the modulation of inflammation and to have pro- and anti-inflammatory actions, respectively. Therefore, SGCs, macrophages, and TG neurons functionally communicate with each other, resulting in the enhancement of the TG neuronal excitability responsible for ectopic orofacial pain. Long after the acceleration of primary afferent activity, TG neurons are sensitized, and TG neuronal activity is further enhanced. These peripheral mechanisms are considered to be involved in the ectopic orofacial pain associated with trigeminal nerve injury and orofacial inflammation (Ji et al., 2016; Batbold et al., 2017; Iwata et al., 2017).

Orofacial noxious information is sent to the Vi and Vc transition zone (Vi/Vc), Vc, and upper cervical spinal cord (C1/C2) via trigeminal nerve fibers (Okamoto et al., 2009; Chiang et al., 2011; Ren and Dubner, 2011). These nuclei have different functions in the processing of orofacial noxious information. Neurotransmitters and neuropeptides are released from the central terminals of the primary afferent neurons and affect the excitability of Vi/Vc, Vc, and C1/C2 nociceptive neurons. After trigeminal nerve injury, the Vi/Vc, Vc, and C1/C2 nociceptive neuronal activities are strongly enhanced, and microglial cells are also activated. Microglial cell activation is reportedly caused by ATP binding to P2X_4_ receptors in microglial cells (Inoue and Tsuda, 2018). Activated microglial cells release BDNF, which binds to the TrkB receptor expressed in second-order neurons in the spinal dorsal horn, resulting in the neuronal hyperexcitability responsible for ectopic orofacial pain (Chiang et al., 2011). Macrophages are also activated and infiltrate in the Vi/Vc, Vc, and C1/C2 regions following trapezius muscle inflammation. The cleavage of FKN from the central terminals of primary afferents innervating the trapezius muscle was enhanced following trapezius muscle inflammation, and microglia in the Vc and C1/C2 were activated via FKN signaling (Kiyomoto et al., 2013). Furthermore, interleukin-1 beta (IL-1β) release was accelerated through p38 phosphorylation followed by microglial activation, and the excitability of the Vc and C1/C2 neurons was enhanced via IL-1β signaling. Trapezius muscle inflammation has been shown to contribute to the hyperexcitability of Vc and C1/C2 neurons receiving inputs from the uninjured orofacial region (Chiang et al., 2011; Kiyomoto et al., 2013; Iwata et al., 2017).

Astrocytes, which are non-neuronal glial cells, are also known to be activated by glutamate uptake with late-onset compared to microglial cells in the Vc. In the central nervous system, these cells also communicate with each other and functionally affect the excitability of Vi/Vc, Vc, and C1/C2 neurons, resulting in the enhancement of the neuronal activity responsible for ectopic orofacial pain (Okada-Ogawa et al., 2009).

It is highly likely that the functional interaction among neurons, immune cells, and glial cells is involved in the enhancement of neuronal excitability in the peripheral and central nervous systems, resulting in the ectopic orofacial pain associated with trigeminal nerve injury and orofacial inflammation. These findings raise the possibility that molecules causing functional changes in neuron–glial interaction following trigeminal nerve injury and orofacial inflammation may be a promising therapeutic target for the treatment of ectopic orofacial pain. In this article, the involvement of functional interaction among TG neurons, immune cells, and glial cells in orofacial pathological pain is reviewed and discussed based on the results of recent animal studies.

## Peripheral Sensitization

### Satellite Glia–Neuron Communication

**FIGURE 1:**
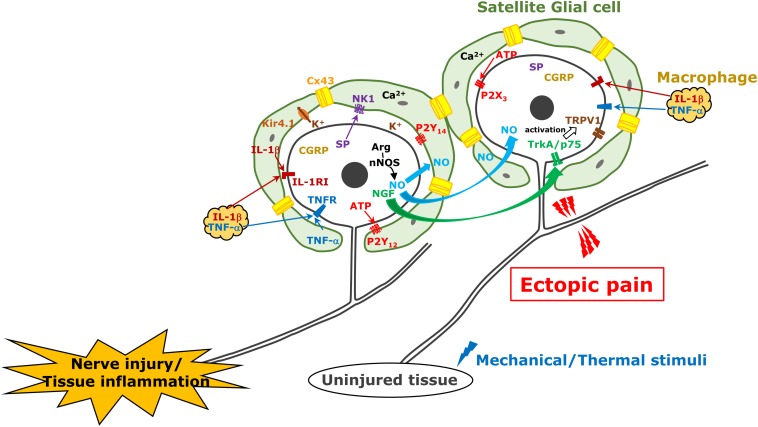
TG neuron, satellite cell, and macrophage communication following trigeminal nerve injury. Following trigeminal nerve injury, TG neurons become hyperactive and various molecules are generated and released from TG neurons; subsequently, satellite glial cells and macrophages are activated. These mechanisms are involved in ectopic orofacial pain associated with trigeminal nerve injury.

Satellite glial cells, which surround the soma of TG neurons, are essential components evoking orofacial pain following nerve injury or inflammation (Hanani et al., 2002; Hanani, 2005; Dublin and Hanani, 2007). Activation of SGCs is characterized by an increase in the expression level of GFAP in SGCs under pathological conditions, whereas this is not observed under non-pathological conditions. GFAP upregulation is observed in the injured trigeminal nerve branch associated with the development of hyperalgesia (Vit et al., 2006; Katagiri et al., 2012). It is believed that neuropeptide such as SP or CGRP is synthesized in TG neurons by various noxious stimuli and is released from neuronal soma as well as peripheral and central terminals, bind their receptors expressed on SGCs, and GFAP expression in SGCs is upregulated after nerve injury or inflammation (Takeda et al., 2005; Vause et al., 2007; Mikuzuki et al., 2017; Zhang et al., 2019). Activated SGCs could release cytokines such as IL-1β in the gap between SGCs and the neuronal soma (Takeda et al., 2007; Lin et al., 2019), that cause acceleration of neuronal excitation. In *in vitro* experiment, SP or CGRP application to the cultured trigeminal SGCs provoke to release some cytokines including IL-1β, TNFα, or IL-6 (Ceruti et al., 2011; Afroz et al., 2019; Zhang et al., 2019). The expression increase of IL-1β in SGCs upregulates voltage-gated Nav1.7 expression in the TG neurons via the cyclooxygenase-2/prostaglandin E2/E prostanoid 2 receptor pathway following temporomandibular joint inflammation (Zhang et al., 2018). It has also been reported in the *in vitro* study that multiple cytokines are released from SGCs by the stimulatory effect of CGRP (Vause and Durham, 2010). Purinergic receptors are also involved in SGCs activation. Increase of P2Y_12_ receptor expression in SGC upregulates GFAP expression in SGCs and enhances TG neuronal excitability following trigeminal nerve injury (Katagiri et al., 2012). Recent findings from a report show activation of P2Y_14_ receptor expressing in trigeminal SGCs upregulates GFAP, IL-1β, and chemokine CCL2 in SGCs via ERK and p38 MAPK activation *in vitro* (Lin et al., 2019), that could cause neuronal excitation (Figure 1).

In TG unlike DRG, neuronal cell bodies are known to be localized in each innervating region (i.e., V1–V3) (Thalakoti et al., 2007). Activation of SGCs surrounding neuronal soma of injured nerve branch spreads into the TG region where the soma of uninjured nerve branches localizes in, that resulting in ectopic orofacial hyperalgesia. The first report was demonstrated in 2007 about neuronal–glial communication through gap junction in the development of sensitization within the TG (Thalakoti et al., 2007). The gap junctions provide direct links and exchange of small molecules and ions between cells under numerous physiological processes. It is believed that this propagation mechanism of activated SGCs is based on gap junctional communication between SGCs. Poulsen et al. (2015) and Feldman-Goriachnik and Hanani (2017) reported that 19–50% of SGCs showed coupling among SGCs around different TG neurons isolated from uninjured mice by dye injection. The former also shows that the dye coupling among SGCs is increased to 44% in neuropathic pain induced by oxaliplatin treatment and the coupling is reversed by gap junction blocker application (Poulsen et al., 2015). Cx43 belongs to the connexin family of proteins in mammals and is not expressed in neurons but in SGCs in the TG (Ohara et al., 2008). The number of Cx43-immunopositive neurons increases in the TG following trigeminal nerve injury in rats, and they are involved in ectopic mechanical allodynia (Kaji et al., 2016). After 3rd branch nerve injury, mechanical hypersensitivity was shown in the upper eyelid skin and whisker pad skin innervated by an uninjured 1st and 2nd branches, respectively, and this sensitization was sustained for at least 2 weeks associated with increase of Cx43 and GFAP protein expression in SGCs surrounding TG neurons innervating uninjured region. The intra-TG injection of the connexin-mimetic peptide attenuates the ectopic hyperalgesia and expressions of both GFAP and Cx43. The detailed relationship between SGCs coupling with the intercellular passage of neuromediators such as Ca^2+^, inositol triphosphate and ATP, and glial activation remains unclear (Chen et al., 2008), although Ca^2+^ quickly passes through gap junctions between glial cells and provokes intercellular calcium waves, which induce increase of glutamate release, reduction of inwardly rectifying potassium channel 4.1 expression, and activation of small-conductance calcium-activated potassium channel 3, increasing neuronal excitability (Vit et al., 2006, 2008; Giugliano, 2009). Cx43 and SGC activation also contributes to ectopic tooth hypersensitivity following inflammation (Komiya et al., 2018). Increase of Cx43 expression and SGC activation are observed under tooth-pulp inflammation. Increased sensitivity to capsaicin in the adjacent tooth pulp to the inflamed one is associated with upregulation of Cx43 and GFAP expression in the SGCs surrounding the soma of TG innervating these teeth via the IL-1β mechanism (Figure 1).

Connexin 43 is believed to play a pivotal role in the propagation of SGC activation mentioned above, which is the basis of ectopic pain. However, increase of gap junction between SGCs in primary cultures of mice TG by the chemotherapeutic agent oxaliplatin (third generation platinum analog) treatment is reported to be independent of Cx43 (Poulsen et al., 2015). More recently, it was reported that there were possible couplings between SGCs, neuron–neuron, and neuron–SGC in TG (Spray et al., 2019). They indicate other connexin types besides Cx43 may be candidates for these couplings.

Nitric oxide is a free radical endogenous gas produced by NOS from L-arginine (Snyder, 1992). There are three types of NOS as follows: nNOS in neurons, iNOS in immunological cells, and endothelial NOS in vascular endothelial cells. NO plays a pivotal role as an endothelial-derived relaxing factor. A unique feature of NO is that it spreads quickly through the cell membrane and its *in vivo* half-life is very short (approximately 1 s) because it is rapidly converted to oxidized nitrogen dioxide. Some evidences have also shown that NO acts as a neuromodulator in the nervous system (Fan et al., 2012). NO activates soluble guanylate cyclase to increase intracellular cGMP concentration. cGMP activates cGMP-dependent protein kinase G, which activates and modulates numerous types of target molecules by phosphorylation (Fan et al., 2012). Freeman et al. (2008) demonstrated NO-proton injection into temporomandibular joint elicit increase of phosphorylated ERK and MAPK in neurons and SGCs located in not only V3 but also V1/V2 regions of the ganglion. Upregulation of nNOS is accompanied by abnormal pain following peripheral nerve injury (Sugiyama et al., 2013). Under the 3rd branch of the trigeminal nerve transection, the mechanical hyperalgesia of the whisker pad skin innervated by the 2nd branch of trigeminal nerve was observed over 6 weeks (Iwata et al., 2001). The expression of nNOS was upregulated in TG neurons innervating the mandibular region, while that in the TG neurons innervating the whisker pad skin did not change even in the presence of mechanical hyperalgesia. The inhibition of nNOS attenuates the mechanical hyperalgesia of the whisker pad skin (Sugiyama et al., 2013) and intra-TG administration of L-arginine causes mechanical hyperalgesia within 2 h after its injection. These results suggest that nNOS expression increases in the neuronal soma of the injured nerve branch; it accelerates NO production and is rapidly diffused into the entire TG, affecting the excitability of uninjured TG neurons. It has also been reported in the *in vitro* study that CGRP in TG neurons is involved in iNOS synthesis in SGCs via MAP kinase signaling (Li et al., 2008; Vause and Durham, 2009). Although it is currently unclear whether NO directly activates TG neurons or indirectly activates via activation of SGCs or macrophages, neuronal NO-protein kinase G signaling can be involved in ectopic orofacial mechanical allodynia (Fan et al., 2012; Figure 1).

### Macrophage–Neuron Communication

At the site of inflammation, nerve injury, or trauma, many types of immune cells including granulocytes, monocytes, lymphocytes, and macrophages are accumulated and activated to initiate tissue repair. Among them, macrophages are richly populated at the site of a lesion and release mediators such as cytokines, chemokines, and neuropeptides (Lee and Zhang, 2012). Nociceptive neuronal hyperexcitation, which leads to intractable pain hypersensitivity, is induced by various cellular and molecular processes related to peripheral inflammation, nerve injury, or trauma. Several mediators released from macrophages bind to their receptors on nociceptive primary afferent neurons, which cause nociceptive neuronal hyperexcitation, which leads to intractable inflammatory and neuropathic pain (Ji et al., 2016). For instance, local-infiltrated macrophages release inflammatory mediators such as TNFα, NGF, LIF, IL-1β, and IL-6 in the inflammatory locus (Zelenka et al., 2005). Moreover, macrophages are also activated by TNFα, which is further conducive to the secretion of inflammatory mediators including IL-1β and TNFα under inflammatory conditions (Mika et al., 2013). TNFR-1 and TNFR-2 are expressed in small DRG neurons, which are considered to be nociceptive primary afferents, TNFα signaling via TNFR-1 and TNFR-2 induces the sensitization of Nav1.8 through protein kinase C activation followed by the promotion of action potential generation and pain hypersensitivity (Guillouet et al., 2011; Leo et al., 2015). The p75 neurotrophin and TrkA receptors act as NGF receptors in nociceptive primary neurons (Meakin and Shooter, 1992). It has been reported that NGF signaling in nociceptive primary afferent neurons enhances the magnitude of the tetrodotoxin-resistant sodium current, decreases in voltage threshold for Nav1.8 activation, and delayed rectifier potassium currents, which accounts for mechanical hypersensitivity in inflamed tissue (Gold et al., 1996; Zhang et al., 2002; Belkouch et al., 2014). Furthermore, the NGF/TrkA complex is internalized in inflamed tissue and transported to the soma, resulting in upregulated expression of Nav1.8 and transient receptor potential vanilloid 1, which plays a crucial role in heat hypersensitivity (Xue et al., 2007; Shinoda et al., 2011). IL-6 signaling also activates the Janus kinase/phosphoinositide 3-kinase signaling pathway in DRG neurons, which results in functional upregulation of transient receptor potential vanilloid 1, followed by cancerous pain hypersensitivity (Fang et al., 2015). Prolonged IL-1β exposure increases DRG neuronal excitability, resulting in pain hypersensitivity by attenuation of the potassium current depending on potassium channel functional alternation *in vitro* (Stemkowski et al., 2015). Small DRG neurons reportedly express the LIF receptor, and pain behaviors are induced by the plantar administration of LIF, while it has been reported that local injection of LIF suppresses inflammatory pain hypersensitivity and IL-1β (Banner et al., 1998; Spofford et al., 2011; Figure 1).

Additionally, activated macrophages do not only infiltrate at the site of inflammation, nerve injury, or trauma but also in the DRG (Ji et al., 2016). Aside from the DRG, the infiltration of non-neuronal immune cells, such as macrophages into the TG, and their activation in the TG are upregulated following orofacial pathogenesis including peripheral trigeminal nerve trauma and orofacial inflammation (Iwata and Shinoda, 2019). Incidentally, most of the macrophages serving as mononuclear phagocytes are supplied from the bone marrow, released from the blood vessels, and migrate into various tissues (Doulatov et al., 2010). Previous studies have indicated that peripheral nerve injury induces increase and activation of resident and proliferated macrophages in the DRG (Lu and Richardson, 1993; Komori et al., 2011; Donegan et al., 2013). The infiltrated and activated macrophages exhibit characteristic morphological structures involving a larger soma and thicker ramifications, which encourages changes in the microenvironment (Harvey et al., 2015). Morphological structural changes indicate their activation (Harvey et al., 2015). Moreover, the migrated macrophages mainly differentiate into two populations that have discrete morphological and functional profiles corresponding to the microenvironment of the migrating sites (Sprangers et al., 2016). Classically activated phenotype called M1 macrophages possess the capacity to secrete numerous pro-inflammatory cytokines and chemokines that participate in the local inflammatory reaction at the early stage. M1 macrophages are activated by interferon-gamma, TNFα, or lipopolysaccharides (Laskin et al., 2011; Brown et al., 2012). In contrast, M2 macrophages possess opposite attributes pertaining to the anti-inflammatory response and the advancement of tissue repair and they are activated by IL-4 and IL-13 (Laskin et al., 2011; Franco and Fernandez-Suarez, 2015). IL-4 and IL-13 signaling in M2 macrophages induces the phosphorylation of signal transducer and activator of transcription 6, and the facilitation of signal transducer and activator of transcription 6-mediated gene transcription is involved in the polarization of macrophages (Waqas et al., 2019). M1 and M2 macrophages are activated at and infiltrate the sites of peripheral nerve trauma and inflammation and also at the sensory ganglion (Komori et al., 2011). The infiltrated and activated macrophages that are known to be differentially involved in the modulation of inflammation release numerous chemical mediators such as cytokines and chemokines. Furthermore, functional cell-to-cell communication between macrophages and TG neurons through some chemical mediators allows for the augmentation of the sensory neuronal excitability in the TG (Iwata et al., 2017). Extended periods of enhancement of sensory neuronal excitability amplify functional cell-to-cell communication leading to neuronal hyperexcitability, resulting in the hyperexcitability of nociceptive neurons in the central nervous system. These plastic changes in cell-to-cell communication in the TG are implicated as a cause of orofacial intractable pain hypersensitivity following orofacial pathogenesis such as trigeminal nerve trauma or inflammation (Chiang et al., 2011). Following peripheral nerve injury, the signaling of CCL2, which is released from the soma of injured neurons via the C–C chemokine receptor type 2 in macrophages, results in macrophage proliferation and its activation in the DRG (Abbadie et al., 2003; Kwon et al., 2015; Liu et al., 2016). Moreover, the increment of CCL2 expression in the soma of TG neurons and the infiltration of macrophages in the TG by peripheral nerve injury never occur under conditions of toll-like receptor 2 deficit, suggesting that the signaling of CCL2 released from injured neurons via toll-like receptor 2 potentiates the abundance of activated macrophage infiltration in the TG after trigeminal nerve trauma (Kim et al., 2011). TNFα in the DRG is considered to derive from macrophages, and the release of TNFα is regulated by intracellular signaling cascades such as ERK and p38 MAPK cascades (Wagner and Myers, 1996; Raghavendra et al., 2003; Wang et al., 2012). Moreover, it has long been known that peripheral nerve injury amplifies SP synthesis in DRG neurons, and synthesized SP was released throughout the DRG (Fu et al., 2013). Some reports have suggested that SP is released into the TG by exocytosis and that it binds the neurokinin 1 receptor expressed in macrophages, which also facilitates TNFα exocytosis from the infiltrated and activated macrophages in the TG through the ERK 1/2 and p38 MAPK signaling pathway (Bardelli et al., 2005; Sun et al., 2008; Matsumoto et al., 2013). Together with these reports, TNFα or SP released by exocytosis from proliferated and activated macrophages following trigeminal nerve injury and enhanced TNFα or SP signaling in the TG lead to TG neuronal hyperexcitability, resulting in orofacial pain hypersensitivity (Batbold et al., 2017; Figure 1).

## Central Sensitization of Central Nervous System Neurons

**FIGURE 2:**
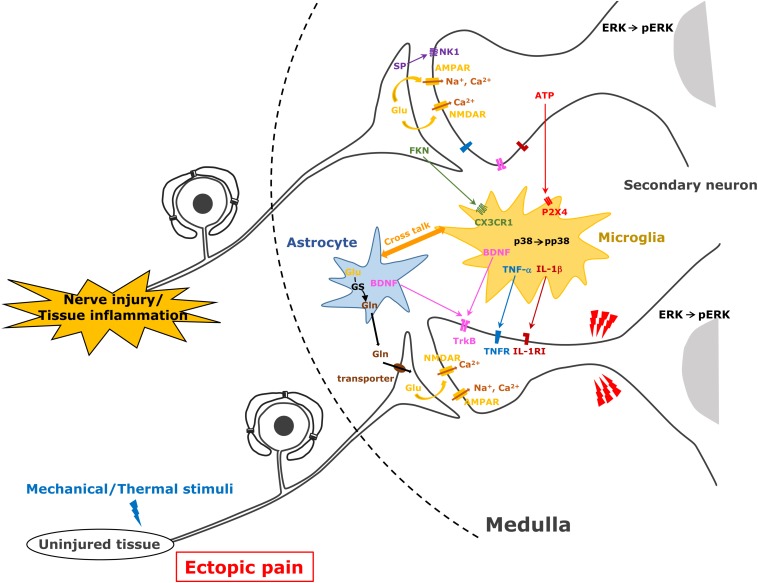
Astrocyte and microglial cell activation following trigeminal nerve injury. After trigeminal nerve injury, astrocytes and microglial cells are activated and various molecules are released from these cells causing further activation of second-order neurons in the trigeminal spinal subnucleus caudalis and upper cervical spinal cord (C1/C2).

Orofacial noxious information is sent to the Vc and C1/C2 with the somatotopic organization via primary afferent TG neurons (Noma et al., 2008). The first branch of the trigeminal nerve projects to the ventral portion of the Vc and C1/C2, the third projects to their dorsal part, while the second branch projects to their middle part. Orofacial noxious stimuli cause activation of nociceptive neurons in these areas according to the regions innervated by each branch of the trigeminal nerve (Iwata et al., 1998; Noma et al., 2008). However, the receptive fields of each nociceptive neuron change their innervation areas, expanding the receptive fields beyond the other branch regions after trigeminal nerve injury or orofacial inflammation (Iwata et al., 1999, 2001). Nociceptive neurons in the Vc and C1/C2 regions are also strongly enhanced after trigeminal nerve injury or orofacial inflammation, resulting in the sensitization of Vc and C1/C2 nociceptive neurons (Iwata et al., 1999; Okada-Ogawa et al., 2009). Various molecules such as neuropeptides, ATP, or neurotrophic factors generated in TG neurons are released from primary afferent terminals and are involved in the modulation of the excitability of Vc and C1/C2 nociceptive neurons (Chiang et al., 2011). Neuropeptides (SP and CGRP), various neurotrophic factors (BDNF and NGF), glutamate, and ATP modulate neuronal activity in uninjured TG neurons as well as injured TG neurons after trigeminal nerve injury and/or orofacial inflammation (Iwata et al., 2011, 2017). NGF and heat-shock protein are released from the peripheral tissues and conveyed to the central nervous system via primary afferent neurons and released from the central terminals (Shinoda et al., 2011; Ohara et al., 2013). Receptors expression to these molecules is strongly enhanced in second-order neurons in the Vc and C1/C2, and these molecules bind to corresponding receptors, resulting in the enhancement of neuronal excitability in Vc and C1/C2 neurons receiving inputs from uninjured orofacial regions (Ren and Dubner, 2008; Iwata et al., 2011, 2017; Figure 2).

It has also been reported that Vi and Vc transition zone (Vi/Vc) has a unique function for orofacial nociception. Many of nociceptive neurons in this area responded to noxious stimulation of the TMJ (Chichorro et al., 2017). Based on c-Fos studies, the number of c-Fos positive neurons did not increase in this area, whereas that was increased in C1/C2 area following noxious stimulation of the TMJ (Bereiter and Okamoto, 2011). Further, noxious responses of TMJ neurons in Vi/Vc altered according to the gender differences, suggesting that TMJ neurons in this area are involved in gender-related TMJ pain. Furthermore, many Vc/C1 neurons respond to dural stimulation, and some of them also respond to bright light stimulation to the eye (Okamoto et al., 2010; Hitomi et al., 2017a). Some bright light-responsive neurons in this area increased in their response magnitude following noxious dural stimulation (Hitomi et al., 2017b). It is also known that many neurons responding to corneal stimulation could be found in this area (Kumar et al., 2018). It is highly likely that nociceptive neurons in this area are involved in multiple sensory functions related to dural pain and corneal pain as well orofacial pain.

After trigeminal nerve injury or orofacial inflammation, various molecules are released from the primary afferent terminals, and these molecules contribute to the activation of microglial cells and astrocytes (Okada-Ogawa et al., 2009; Shibuta et al., 2012). ATP and glutamate are essential molecules for the activation of microglial cells and astrocytes, respectively. P2X_4_ receptor expression is known to be significantly enhanced in microglial cells in the spinal dorsal horn after sciatic nerve injury (Inoue and Tsuda, 2018). After peripheral nerve injury, the accumulation of ATP released from primary afferent terminals or blood vessels is strongly accelerated in the spinal dorsal horn, and ATP binds to the P2X_4_ receptor expressed in the microglial cells (Inoue and Tsuda, 2018). As P2X_4_ is a cation permeable purinergic receptor, ATP binding to the P2X_4_ receptor causes activation of the intracellular signal cascade (Inoue and Tsuda, 2018). Ca^2+^ influx causes p38 phosphorylation and various proteins are produced. Cleaved FKN from the primary afferent terminals is also known to be involved in the activation of microglial cells after peripheral nerve injury or inflammation. FKN binds to the FKN receptor expressed in the microglial cells involved in microglial cell activation (Kiyomoto et al., 2013). FKN binding to its receptor also causes p38 phosphorylation in microglial cells, resulting in the production of various cytokines (Kiyomoto et al., 2013).

Activated microglial cells produce various pro-inflammatory cytokines (IL-1β, TNFα, and IL-6), BDNF, and ATP and activated astrocytes produce CCL2, glutamine, and NF-κB and release these molecules under the trigeminal neuropathic state (Iwata et al., 2011, 2017; Goto et al., 2016). Conversely, after orofacial inflammation, activated microglial cells produce various cytokines and BDNF, and activated astrocytes produce BDNF and glutamine (Chiang et al., 2011). These molecules are considered to be involved in modulation of Vc and C1/C2 nociceptive neuronal activity (Figure 2).

Astrocytes are also activated following trigeminal nerve injury or orofacial inflammation in the Vc and C1/C2 (Okada-Ogawa et al., 2009; Tsuboi et al., 2011). Glutamine–glutamate shuttle in the activated astrocytes is considered a key mechanism involving modulation of neuronal activity in the Vc and C1/C2. After the activation of astrocytes, glutamine is produced by the action of glutamine synthetase in astroglial cells and released from these cells (Chiang et al., 2011; Figure 2). Glutamine is transferred from the primary afferent terminals of the trigeminal nerve via the glutamine transporter, and then glutamate release is accelerated, resulting in the hyperactivation of Vc and C1/C2 neurons. Activated astrocytes also release CCL2 and NF-κB, and these molecules are also known to be involved in the modulation of the excitability of Vc and C1/C2 nociceptive neurons (Iwata et al., 2017).

There is a large number of interneurons in the Vc and C1/C2 and many of them are classified as GABAergic or glycinergic interneurons involved in excitability decrease in the vicinity of GABAergic neurons (Okada-Ogawa et al., 2015). After trigeminal nerve injury, the number of inhibitory interneurons in the Vc and C1/C2 regions is reduced, resulting in enhanced excitability of nociceptive neurons (Okada-Ogawa et al., 2015). Furthermore, KCC2 downregulation occurs in Vc and C1/C2 neurons, and the chloride ion is accumulated in nociceptive neurons, resulting in the enhancement of nociceptive neuronal activity by inhibitory interneurons acting as excitatory.

## Involvement of Descending Modulation

Severe inflammation in the orofacial region causes strong activation of the descending pathways as well as ascending noxious pathways (Gu et al., 2011; Okubo et al., 2013). The RVM in the reticular formation is a critical area involved in the descending modulatory system (Lau and Vaughan, 2014; Martins and Tavares, 2017). Three types of neurons, ON cells, OFF cells, and neutral cells, are differentially involved in the modulation of nociceptive neurons in the Vc and C1/C2 (Hitomi et al., 2017a). Many serotonergic neurons that exist in the RVM are involved in the modulation of Vc and C1/C2 nociceptive transmission. The descending system appropriately alters the excitability of nociceptive neurons in the Vc and C1/C2 under normal conditions, contributing to the sensory-discriminative aspect of orofacial pain. After orofacial deep tissue inflammation, microglial cells are activated in the RVM, and the excitability of ON cells further accelerating, and Vc and medullary neuronal activities becoming strongly enhanced, resulting in orofacial hyperalgesia (Wei et al., 2008). These findings suggest that the glial cell activation in RVM is also involved in the modulation of descending inhibitory and excitatory systems associated with orofacial inflammation.

## Author Contributions

MS, AK, YH, and KI wrote the manuscript. All authors have read and approved the final manuscript.

## Conflict of Interest

The authors declare that the research was conducted in the absence of any commercial or financial relationships that could be construed as a potential conflict of interest.

## Abbreviations

ATP: adenosine triphosphate
BDNF: brain-derived neurotrophic factor
CCL2: chemokine C–C motif ligand 2
CGRP: calcitonin gene-related peptide
DRG: dorsal root ganglion
ERK: extracellular signal-regulated kinase
FKN: fractalkine
GFAP: glial fibrillary acidic protein
iNOS: inducible nitric oxide synthase
LIF: leukemia inhibitory factor
MAPK: mitogen-activated protein kinase
Nav: voltage-gated sodium channel
NGF: nerve growth factor
nNOS: neuronal nitric oxide synthase
NOS: nitric oxide synthase
RVM: rostro-ventral medulla
SGC: satellite glial cell
SP: substance P
TG: trigeminal ganglion
TNF: tumor necrosis factor
TNFR: TNF receptor
Vc: trigeminal spinal subnucleus caudalis
Vi: trigeminal subnucleus interpolaris.
